# Effect of Ta Content on the Microstructure and Properties of NiTiTa Functional Coatings In Situ Synthesized by Directed Energy Deposition

**DOI:** 10.3390/ma18225255

**Published:** 2025-11-20

**Authors:** Sansan Ao, Yawei Xing, Shaozhu Liu, Xinde Zuo, Yang Li

**Affiliations:** 1 School of Materials Science and Engineering, Tianjin University, Tianjin 300350, China; xingyw123@163.com (Y.X.); zuoxinde@tju.edu.cn (X.Z.); liyang86@tju.edu.cn (Y.L.); 2 The National Key Laboratory of Particle Transport and Separation Technology, Tianjin 300350, China; 3 PipeChina North Pipeline Company, Langfang 065000, China; shaozhuliu@126.com

**Keywords:** directed energy deposition, NiTiTa coating, microstructure, corrosion resistance, X-ray visibility

## Abstract

In this study, surface alloying technology based on Gas Tungsten Arc Welding (GTAW) was used to synthesize in situ NiTiTa coatings on a NiTi substrate using commercially pure Ta foils. The influence of different Ta contents (0.91, 1.42, and 2.91 at.%) on the microstructure, phase formation, hardness, corrosion resistance, and X-ray visibility of the prepared coatings were systematically studied. These results show that the NiTiTa coatings fabricated by GTAW were free of microcracks with good surface quality and superior adhesion to the NiTi substrate. The NiTiTa coatings are mainly composed of columnar austenitic NiTi (B2), and martensitic NiTi (B19’) with (Ti, Ta)2Ni precipitating at the grain boundaries. The proportion of B19’ martensite and the Ta content dissolved in the NiTi matrix increases with the increasing addition of Ta. In addition, β-Ta appeared in the coating formed with 1.42 at.% Ta and precipitated abundantly when the Ta amount was increased to 2.91 at.%. Changes in phase composition and secondary phases lead to a decrease in the material nanohardness. To simulate the body fluid environment, corrosion tests were conducted in Hank’s solution at a rate of 0.5 mV/s. Electrochemical tests show that the NiTiTa coatings exhibit superior corrosion resistance, where the corrosion potential, Ecorr, increased with increasing Ta content. The enhanced X-ray visibility of the newly formed coatings was also revealed. This work provides a cost-effective method for in situ synthesis of NiTiTa coatings on NiTi alloys, highlighting its potential for improving the corrosion resistance and X-ray visibility of NiTi shape memory alloys.

## 1. Introduction

NiTi shape memory alloys (SMAs) have been widely used in biomedical fields owing to their remarkable shape memory effect (SME), superelasticity (SE), excellent biocompatibility, and corrosion resistance [1,2]. Previous studies have shown that the main limitations associated with the safe development of NiTi SMAs for biomedical applications are mainly related to potential biocompatibility issues [3,4]. Ni ions may sometimes be released, and these are harmful to the human body [5]. Additionally, the relatively low atomic number and low density of these alloys result in poor X-ray visibility, which makes it challenging to detect small-size devices made of NiTi, such as stents [6,7]. To solve such problems, surface coatings with corrosion-resistant and radiopaque materials have been demonstrated to be one of the most promising techniques among the various NiTi surface modification approaches reported [8].

Tantalum (Ta), a semi-precious refractory metal with a melting point of 2996 °C, has attracted increasing attention in the metallic biomaterials field due to its excellent corrosion resistance, histocompatibility, and high radiopacity [9]. It has become an important material for the surface coating of NiTi alloys. For decades, different processes have been used to prepare Ta coatings on NiTi alloy surfaces, including magnetron-sputtering [10], plasma immersion ion implantation deposition [11], and multi-arc ion plating [8]. Still, these traditional fabrication methods are inefficient, which may limit their applications. In addition, for the direct use of pure Ta as a coating, NiTiTa ternary alloys with proper addition of Ta have been proven to have higher corrosion resistance and X-ray visibility than conventional binary NiTi alloys [12]. Meanwhile, other elements also exhibit distinct strengthening effects on NiTi alloys. For instance, Niu et al. incorporated Nb into NiTi alloys to enhance their corrosion resistance [13]. Wu et al. added Cu to improve both compressive strength and antibacterial properties [14].

Ta exhibits unique characteristics among the numerous elements influencing NiTi alloys. Gong et al. found that controlled additions of Ta (less than 3 at.%) could be dissolved in the matrix, and the extra Ta enabled the formation of (Ti, Ta)2Ni and β-Ta secondary phases [15,16]. Cai et al. studied as-casted NiTiTa alloys with different Ta contents (from 1.3 to 15 at.%) and found that the increase in Ta addition improved the material radiopacity. However, when the Ta content was higher than 5 at.%, brittle (Ti, Ta)2Ni formed, which would reduce the ductility and fatigue performance of the alloy [12,17]. Moreover, the increase in Ta also affects the surface roughness [18] as well as the phase transformation temperatures [19].

Despite the potential to improve the functional properties of NiTi alloys, no results related to the radiopacity of NiTiTa coatings have been reported so far. The high melting point of Ta restricts the in situ synthesis of NiTiTa alloys by mainstream methods. For example when using a laser source for cladding Ta onto a NiTi substrate, the instantaneous high temperature generated by the short-duration laser cannot provide sufficient heat to fully melt the Ta powder [20].

Various technologies have been successfully applied in surface alloying, such as laser powder deposition (LPD) [21], plasma transferred arc (PTA) [22], and Gas Tungsten Arc Welding (GTAW) [23]. Rynkus et al. employed electrophoretic deposition (EPD) technology to deposit SiO_2_ on NiTi alloys, with the research findings highlighting the coating’s susceptibility to passivation corrosion [24]. Yu et al. employed laser powder bed fusion (LPBF) technology to fabricate NiTi alloy specimens, revealing issues such as weak corrosion resistance and low dimensional accuracy [25]. Wang et al. synthesized a protective NiTi coating in situ on a Ti6Al4V substrate using separate pure Ni and Ti wires as the feedstock, which confirmed the feasibility of in situ synthesis of NiTi-based alloys via GTAW [23]. Saroj et al. fabricated TiC-reinforced Inconel 825 alloy matrix composite coatings by a GTAW cladding process [26], demonstrating that this technology has the potential to clad refractory metals due to sufficient heat input [27]. Overall, LPBF has a narrow post-processing window, with EPD coatings exhibiting limited durability and interfacial bonding, making it difficult to ensure consistency in small components. In contrast, GTAW offers simplicity, low cost, suitability for small-part joining, and controllable heat input, demonstrating its advantages for engineering-scale manufacturing of NiTi [28].

In the present study, GTAW technology was employed for the first time for in situ synthesis of NiTiTa coatings onto a NiTi substrate by prelaying pure Ta foils. The use of Ta foils avoids the high cost of custom Ta wires and also allows the Ta content to be adjusted by changing the width or thickness of the Ta foils. This work provides a cost-effective solution for in situ synthesis of the NiTiTa coatings to enhance the corrosion resistance and X-ray visibility of NiTi alloys. The effects of Ta content on the microstructure and mechanical properties of NiTiTa coating properties were also systematically investigated.

## 2. Materials and Methods

### 2.1. GTAW-Based Surface Alloying Setup

**Figure 1:**
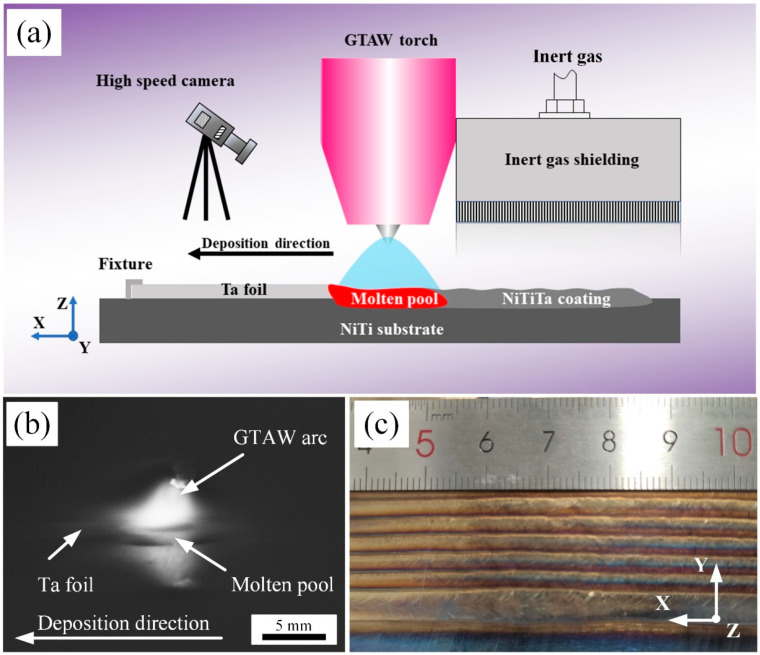
(**a**) Schematic diagram of the surface alloying setup; (**b**) high-speed camera picture during the cladding process; (**c**) macromorphology of the fabricated NiTiTa coatings.

The NiTiTa coatings were deposited by a surface alloying system (detailed in Figure 1a), which mainly consisted of a GTAW machine WSME-315 (Aotai Electric Co., Ltd., Jinan, China) using cerium tungsten with a diameter of 2.5 mm as the negative electrode, an inert gas trailing shield, a travel device, and a high-speed camera Cyclone-2-2000-M (Optronis Co., Ltd., Kehl, Germany), where the camera was used to monitor the melting of Ta and the evolution of the molten pool. A NiTi substrates with a size of 200 mm × 100 mm × 10 mm and Ta foils with a thickness of 0.1~0.2 mm were selected as raw materials in this study. The NiTiTa coatings could be in situ metallurgically synthesized by prelaying pure Ta foils onto the surface of NiTi substrates. The Ta content could be adjusted by changing the width and thickness of the Ta foils. High-purity argon gas (99.999%) was used for both GTAW torch and trailing shield. Preliminary experiments indicated that coating exhibits optimal surface finish when the electrode offset between two passes is 3 mm. Therefore, the offset was set to 3 mm for this experiment. The detailed chemical composition of the raw materials and the main parameters used during deposition are presented in Table 1 and Table 2, respectively. Three different sizes of Ta foils were selected to fabricate the NiTiTa coatings with three different Ta contents, as shown in Table 3. The corresponding Ta contents measured by EDS are 0.91, 1.42, and 2.91 at.%, hereafter referred to as NiTi-0.91Ta, NiTi-1.42Ta, and NiTi-2.91Ta samples, respectively. The average measurement error of the elemental composition is less than 0.25 at.%.

### 2.2. Macro Morphologies and Microstructure Characterization

Three-dimensional laser confocal microscopy VK-X1050 (Keyence (China) Co., Ltd., Shanghai, China) was used to observe the surfaces of NiTi substrates and NiTiTa coatings under a magnification of 5. The metallographic specimens were cut using a wire-electrode cutting. Then the samples were mounted in epoxy, ground with silicon carbide sandpaper from 80# to 2000#, and etched for 30 s using a HF:HNO_3_:H_2_O (3:5:100 mL) solution after mechanical polishing. The samples were cleaned using ultrasonic cleaning with anhydrous ethanol and then air-dried. The microstructure of the NiTiTa coatings was evaluated, combining optical microscopy (OM, Olympus microscope) and scanning electron microscopy Sigma 500 (Zeiss Corp., Osnabruck, Germany), with an energy-dispersive spectrometer (Oxfords Instruments Corp., Oxfordshire, UK) for elemental analysis. The phase composition of NiTiTa coatings was studied by X-ray diffraction D8 Advanced (Bruker Corp., Karlsruhe, Germany) with a radiation of Cu Kα and a step size of 0.01°, and the angle range of 20–90° was selected.

### 2.3. Nanoindentation Testing

The hardness of the NiTiTa coatings was obtained using a nanoindenter, TI-Premier, (Hysitron Inc., Eden Prairie, MN, USA) with a Berkovich diamond indenter. Nanoindentation tests were performed on polished surfaces of the NiTi substrate and NiTiTa coatings with a maximum applied load of 6000 mN, a loading/unloading rate of 100 mN/s, and a dwell time of 10 s. The nanoindentation hardness, H, of the NiTiTa coatings was calculated as the ratio of the indentation force to the projected contact area on the surface of the samples.

### 2.4. Electrochemical Measurements

Potentiodynamic polarization tests were used to assess the electrochemical corrosion behavior of the fabricated materials. A classical three-electrode electrochemical system (LK3200A) was used. The saturated calomel electrode (SCE), the platinum foil, and the prepared samples with dimensions of 10 mm × 10 mm were used as the reference electrode, counter electrode, and working electrode, respectively. Hank’s balanced salt solution (HBSS) was maintained at 37 °C during testing with a constant thermal bath to simulate body fluids. Hank’s solution is a physiological saline solution that mimics body fluids. Its primary components include sodium chloride, phosphates, sodium bicarbonate, potassium sulfate, and others, with a pH typically ranging between 7.2 and 7.4. Before each experiment, all samples were immersed in the solution for 30 min to reach a stable open circuit potential (OCP). The polarization curves were measured separately from −1000 mV to 550 mV at a scanning rate of 0.5 mV/s. X-ray photoelectron spectroscopy, model AXIS Supra (Kratos Analytical Ltd., Kratos, UK) was used to determine the surface chemical composition and state of the NiTiTa coatings after electrochemical corrosion.

### 2.5. X-Ray Visibility Tests

X-ray tests were performed by a XDX-AZ350 (Shanghai Anzhu Photoelectric Technology Co., Ltd., Shanghai, China) X-ray source to study the X-ray visibility of NiTi substrates coated with NiTiTa coatings. Specimens were cut into a size of 4 mm × 4 mm × 3.5 mm, and the accelerating voltage, current, and exposure time were 60 kV, 1 mA, and 2 s, respectively. In order to quantitatively describe the radiopacity of the NiTi substrate and the NiTiTa coatings, Adobe Photoshop CS6 software 2021 was used to measure the brightness of each sample under the Hues, Saturation, and Brightness (HSB) mode, and five equidistant points along the length of each X-ray image were selected to calculate the average brightness value.

## 3. Results and Discussion

### 3.1. Macro Morphologies

The picture captured by the high-speed camera shown in Figure 1b details that the Ta foil was completely melted by the GTAW arc and was directed into the molten pool during deposition. From Figure 1c, it can be observed that the NiTiTa coating shows a continuous and uniform cladding to the NiTi substrate without macroscopically visible defects, such as spatter.

**Figure 2:**
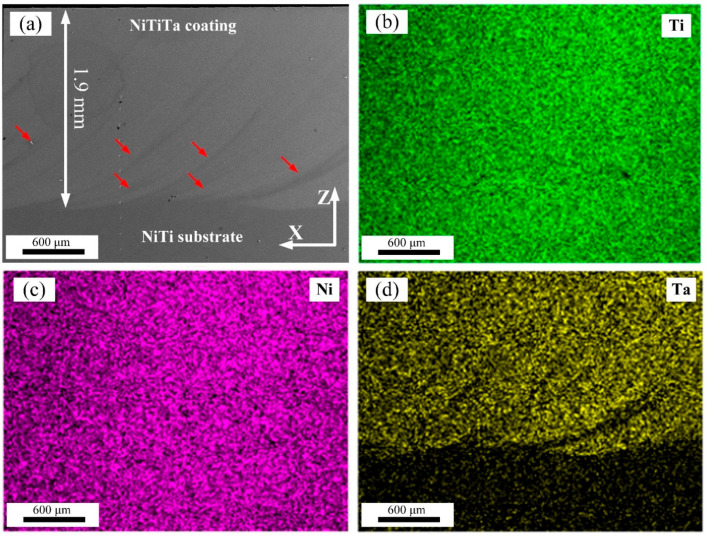
(**a**) Macromorphology of the cross section of NiTi-1.42Ta coating; (**b**–**d**) EDS mapping of Ti, Ni, and Ta, respectively. The red arrows are extending from the substrate to the inside of the coating.

Figure 2a presents the SEM image of the cross section of the NiTi-1.42Ta coating. No macroscopic defects such as pores or cracks were observed. The thickness of the coating is about 1.9 mm, which is significantly thicker than previously reported micron-sized Ta coatings [8,10,11], thus demonstrating the high efficiency of GTAW technology for coating cladding. In addition, many arc-shaped regions (marked with red arrows) are extending from the substrate to the inside of the coating, which was caused by the complex flow and rapid solidification of the molten pool. The corresponding EDS mappings of Ti, Ni, and Ta elements (refer to Figure 2b–d) confirm that the arc-shaped regions have lower Ta content, and Ni, Ti, and Ta elements are uniformly distributed in other regions of the coating.

**Figure 3:**
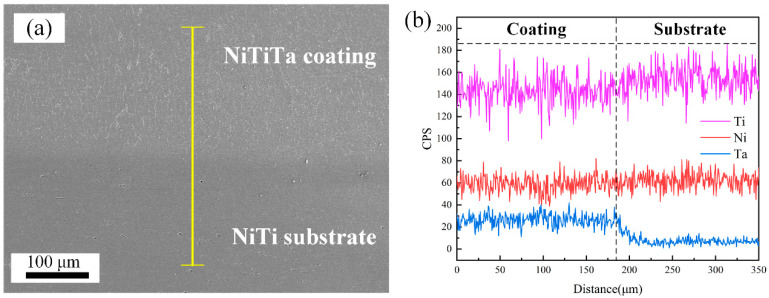
(**a**) Macromorphology of the transition region; (**b**) EDS line scan profile along the yellow line in (**a**).

EDS line scan results of the bonding area between the NiTi substrate and the NiTiTa coating are detailed in Figure 3. A transition interface with a width of about 40 μm, when there is some Ta diffusion into the NiTi substrate, can be observed. This is believed to effectively enhance the bonding strength between the NiTi substrate and the NiTiTa coating [29]. It should be emphasized that the dimension of this transition interface, as well as the diffusion length of the Ta into the NiTi substrate, will depend on the selected process parameters used during deposition.

**Figure 4:**
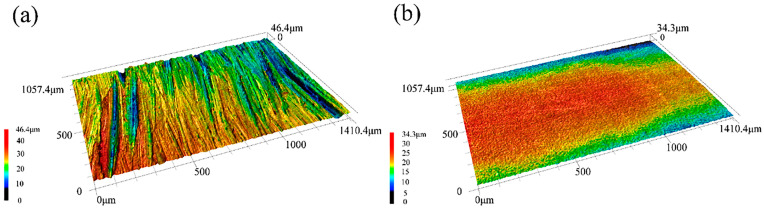
Laser confocal images of the NiTi substrate (**a**) and the NiTiTa coating (**b**).

Surface finish is an important property that affects the behavior of mating contact surfaces. Laser confocal tests were used to analyze the topographical features of the NiTi substrate and NiTiTa coating surfaces at the micro scale, as shown in Figure 4. The NiTiTa coating shows an acceptable surface finish with a maximum surface height of 34.3 μm, while the machined NiTi substrate exhibits a maximum height of 46.4 μm, which shows significant grindings and scratches on the surface. Obviously, the surface finish of the prepared NiTiTa coating is substantially improved compared to the NiTi substrate. These results differ from the poor surface quality of wire and arc additive manufacturing (WAAM) reported in previous works [30]. A major reason is that the application of the Ta foils avoids droplet transfer during traditional wire deposition process. The Ta foils were completely melted under the action of the electric arc and were directed into the molten pool (shown in Figure 1b), ensuring a continuous and smooth surface quality. In addition, it has been reported that increasing the Ta percentages in NiTi alloys helps in reducing the material surface roughness [18].

### 3.2. Microstructural Characterization

**Figure 5:**
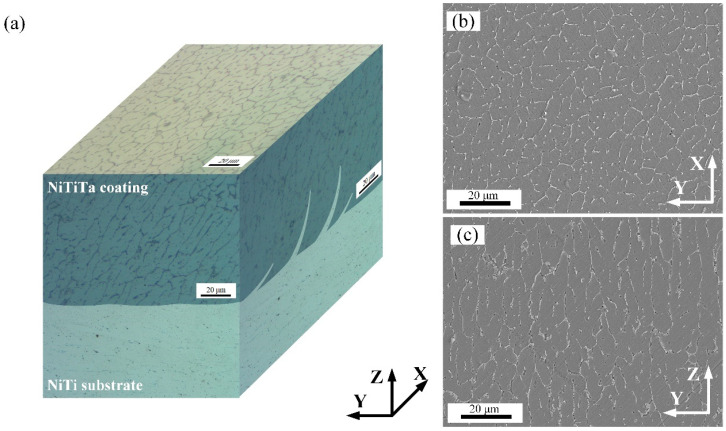
(**a**) Three-dimensional representation of the microstructure characteristics of the NiTiTa coating; SEM images of the NiTiTa coating (**b**) surface and (**c**) cross section.

Figure 5a presents the 3D reconstructed microstructure of the NiTiTa coating on the NiTi substrate. This technique has also been employed in other studies [31]. The coatings are mainly composed of a large number of columnar grains, and equiaxed grain structures can be observed from the coating surface. These grain structures are also confirmed by SEM imaging (shown in Figure 5b,c), and enable the visualization of precipitates at the grain boundaries. The development of columnar grains is related to the large thermal gradient caused by the heat dissipation of the substrate during deposition [32]. The columnar grains grew along the heat dissipation direction and converged to the coating surface, eventually forming an equiaxed grain structure at the top of the structure.

**Figure 6:**
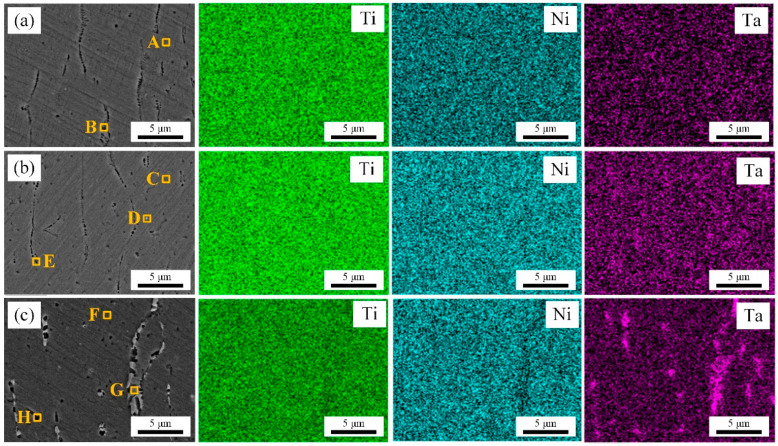
SEM images and corresponding elemental EDS mappings of different coatings: (**a**) NiTi-0.91Ta; (**b**) NiTi-1.42Ta; and (**c**) NiTi-2.91Ta. A–H are the location of the selected points.

SEM and EDS analyses were performed on enlarged cross sectional areas to further investigate the microstructure and the composition distribution of NiTiTa coatings with different Ta contents. The results are shown in Figure 6. Table 4 lists the compositional analysis of the points (A~G) marked in Figure 6. For the NiTi-0.91Ta coating (Figure 6a), oval precipitates (marked with B) can be observed distributed at the grain boundaries, and the corresponding EDS mappings indicate that the Ni, Ti, and Ta elements are uniformly distributed in the matrix. Combined with the EDS point analysis in Table 4, the matrix (marked with A) corresponds to the NiTi phase with 0.87 at.% Ta solid solution, while the precipitate with a (Ti + Ta)/Ni ratio of 1.73 is highly likely to be the (Ti, Ta)_2_Ni phase. There is no obvious difference in the microstructure of the NiTi-1.42Ta and NiTi-0.91Ta coatings, but individual white precipitates (marked with D) near the grain boundaries of NiTi-1.42Ta coating can be observed in Figure 6b. Based on EDS point analysis, these precipitates exhibit a Ta content as high as 65.56 at. %, which is likely β-Ta phase. In addition, the Ta content in the matrix of NiTi-1.42Ta coating increased to 1.34 at.%. In contrast, the microstructure of the NiTi-2.91Ta coating changed significantly. Besides the oval (Ti, Ta)2Ni phase (marked with H), a large number of irregular white precipitates (marked with G) appeared at the grain boundaries, as shown in Figure 6c. The elemental mappings reveal that the white phase is rich in Ta. Combined with EDS point analysis, the white precipitates are highly likely to be the β-Ta phase, and the tantalum content in the matrix coating has increased to 2.88 at.%.

Through quantitative analysis of the selected regions (A~H) in Figure 6, it can be concluded that the NiTiTa coatings consist of (Ti, Ta)2Ni precipitates and NiTiTa solid solution when the Ta content is relatively low. With the increase in Ta addition, the amount of dissolved Ta in the NiTi matrix increases, and the Ta-rich β-Ta phase starts to precipitate at the grain boundaries, which is similar to what was previously reported for as-cast NiTiTa alloys [33].

### 3.3. Phase Identification

**Figure 7:**
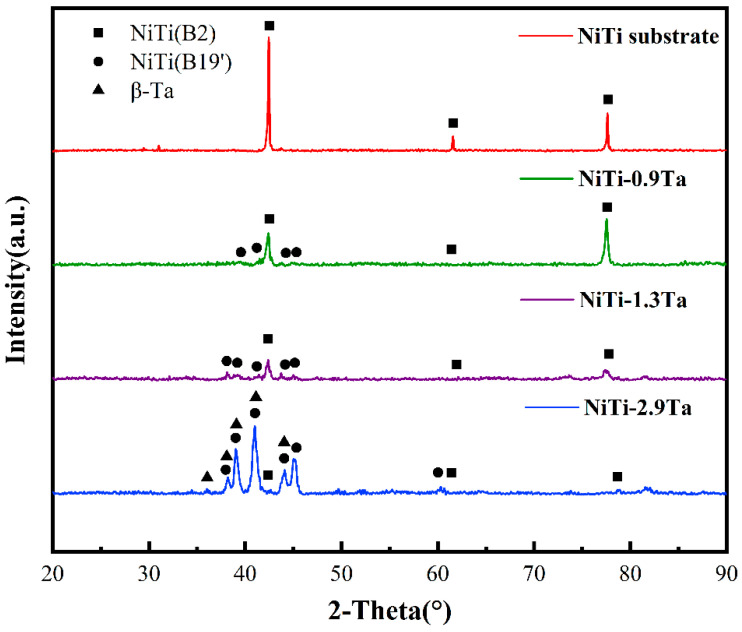
XRD patterns of the NiTi substrate and NiTiTa coatings with different Ta contents.

The phase constituent is one of the most critical factors controlling coating performance. Figure 7 details the XRD patterns of the NiTi substrate and NiTiTa coatings with different Ta contents. Diffraction peaks corresponding to the (110) B2, (200) B2, and (211) B2 planes indicate that the NiTi substrate is fully austenitic. In contrast, the NiTi-0.91Ta coating transformed into a mixture of both B2 austenite and B19’ martensite. The intensity of the B19’ martensite peaks in the NiTi-1.42 coating increased, indicating an increase in the proportion of this phase, with a reduction in the amount of B2 austenite. For the NiTi-2.91Ta coating, the diffracted intensity of the B2 austenite phase peaks is relatively weak compared to those of the B19’ martensite phase, indicating the dominance of martensite in the coating. Notably, a small number of β-Ta peaks are observed, confirming the formation of the β-Ta phase in the NiTi-2.91Ta coating, which further confirms the correctness of the previous EDS analysis. However, contrary to the EDS results, no significant (Ti, Ta)2Ni diffraction peaks were observed in the XRD results of the three samples, which may be due to the low content of (Ti, Ta)2Ni and the particle size being at the nano-scale level.

The addition of Ta will affect the phase structure and volume fraction of the existing phases in the material, thereby suggesting a change in the phase transformation temperatures of the fabricated materials. Ma et al. [19] found that the increased Ta content would decrease the Ni/Ti ratio, eventually resulting in an increase in the martensite transformation temperatures. The martensitic phase in NiTiTa coatings is believed to benefit the biomedical applications due to its superior compression performance [34].

### 3.4. Nanoindentation

**Figure 8:**
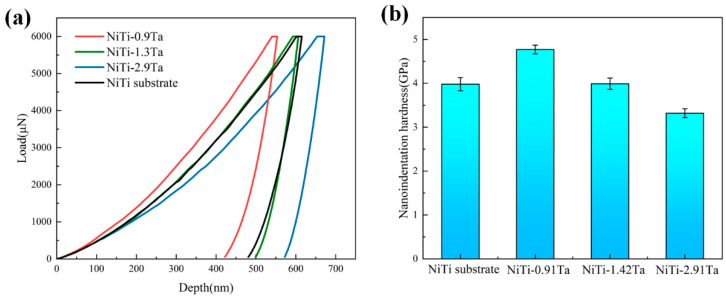
Nanoindentation curves (**a**) and corresponding hardness histograms (**b**) of the NiTi substrate and NiTiTa coatings with different Ta contents.

To further study the effect of Ta contents on the mechanical properties of NiTiTa coatings, nanoindentation load displacement curves of NiTiTa coatings and NiTi substrate were obtained, as shown in Figure 8a. The calculated results of hardness are displayed in Figure 8b.

It is obvious that the hardness of the NiTi-0.91Ta coating is higher than that of the NiTi substrate, but with the increased addition of Ta, the coating hardness decreased gradually. These results further support the XRD analysis and previous microstructural characterization. First, for the NiTi-0.91Ta coating, the addition of the Ta element resulted in solid solution strengthening. Moreover, the formation of brittle (Ti, Ta)2Ni precipitates in the NiTi-0.91Ta coating also significantly increased the material hardness. These two factors led to a higher hardness value of the NiTi-0.91Ta coating compared to the original NiTi substrate. With the increase in Ta content in the coating, the reduction in nanoindentation hardness is mainly attributed to the increased NiTi martensite phase fraction, as the modulus of the NiTi martensite phase (21–69 GPa) is known to be lower than that of the NiTi austenite phase (70–110 GPa) [35]. In addition, precipitation of the soft β-Ta phase in NiTi-1.42Ta and NiTi-2.91Ta coatings led to a further decrease in hardness. Nevertheless, unlike the previously reported significant hardness difference brought by Ta-coated NiTi alloys [29], the hardness of the in situ-synthesized NiTiTa coatings was close to that of the NiTi substrate, which is believed to avoid possible surface performance issues and benefit practical applications.

### 3.5. Electrochemical Behavior

**Figure 9:**
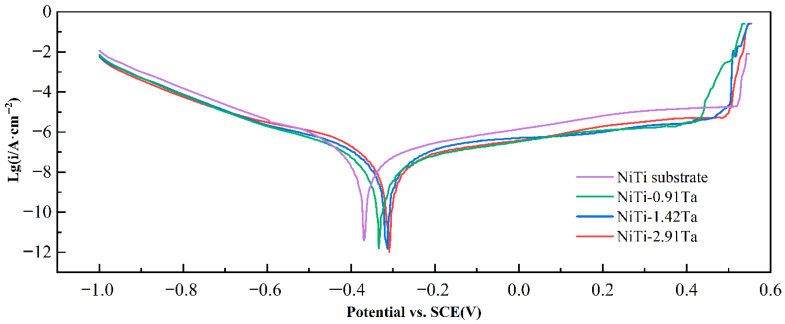
Potentiodynamic polarization curves of the NiTi substrate and NiTiTa coatings with different Ta contents in Hank’s solution at 37 °C.

**Figure 10:**
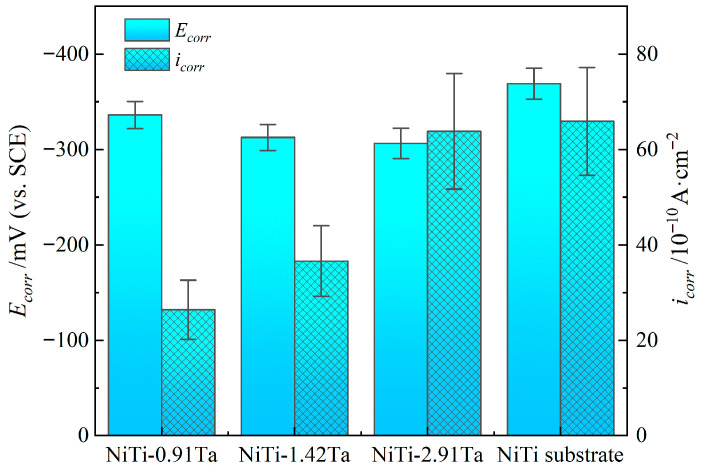
The main electrochemical parameters.

Figure 9 details the potentiodynamic polarization curves of the different NiTiTa coatings and of the NiTi substrate in Hank’s solution. All NiTiTa coatings exhibit a wide passive region with no apparent rapid increase in the anodic current density, indicating that their passive films are able to effectively protect the NiTi substrate from pitting corrosion. The corrosion potential (*E_corr_*) and the corrosion current density (*i_corr_*) of NiTiTa coatings and NiTi substrate are calculated by Tafel extrapolation for comparison, and these data are shown in Table 5 and Figure 10.

NiTiTa coatings show higher *E_corr_* and lower *i_corr_* than the NiTi substrate, and the *E_corr_* slightly increases with the increase in Ta content. It can be inferred that Ta exhibits good chemical stability. The addition of Ta forms a thin film in the electrolyte, which effectively inhibits corrosion reactions, thereby increasing the corrosion potential *E_corr_* and decreasing *i_corr_*. However, at higher Ta concentrations, the corrosion rate recovers [6,7]. The *i_corr_* values of NiTi-0.91Ta, NiTi-1.42Ta, and NiTi-2.91Ta coatings are 26.4 × 10^−10^, 36.6 × 10^−10^, and 63.8 × 10^−10^ A·cm^−2^, respectively. The existence of the β-Ta phase in the NiTiTa coatings may be the cause of this phenomenon [29]. This is because high Ta concentrations may compromise the integrity of the passivation film, leading to film instability. High concentrations of β-Ta may induce localized fragility in the film, causing localized fractures or detachment. This exposes the surface to the corrosive medium, paradoxically increasing the corrosion rate.

**Figure 11:**
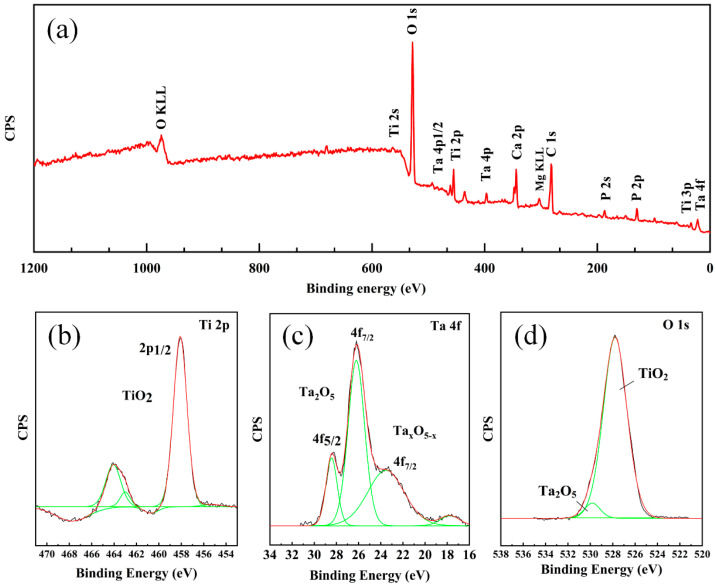
(**a**) XPS spectra of NiTiTa coating after electrochemical corrosion; high-resolution XPS spectra (**b**) Ti 2p, (**c**) Ta 4f, and (**d**) O 1s.

To illustrate the corrosion resistance mechanism of the NiTiTa coatings, XPS testing was performed on the surface of the NiTi-2.91Ta coating after electrochemical corrosion. Figure 11a is the XPS measurement spectrum, where the dominant O, Ti, and Ta elements with residual Mg, Ca, P, and C elements can be observed, indicating the generation of the passivation film. Mg, Ca, P, and C elements are related to the remaining Hank’s solution on the surface of the passivation film. Analysis of the high-resolution spectra of Ti 2p (Figure 11b) and Ta 4f (Figure 11c) demonstrated the formation of Ta and Ti oxides. Specifically, Ti 2p_1/2_ at 458.1 eV confirms the formation of TiO_2_, and the Ta 4f_7/2_ line position at 26.3 eV is typical of the Ta chemical state in Ta_2_O_5_. In addition, the existence of a small amount of non-stoichiometric Ta oxide (Ta_2_O_5_−X) is proven by the Ta 4f_7/2_ line position at 23.4 eV. These results are also confirmed by the spectra of O 1s in Figure 11d. The XPS results indicate that the passivation film of the NiTiTa coatings is dominated by TiO_2_ and contains a small amount of Ta_2_O_5_ and Ta_2_O_5_−X. Therefore, the improved corrosion resistance of NiTi alloys coated with NiTiTa can be attributed to the formation of Ta-containing oxides, which have been reported to increase the electric resistivity and enlarge the thickness of passivation films of NiTi alloys [36].

### 3.6. X-Ray Visibility

**Figure 12:**
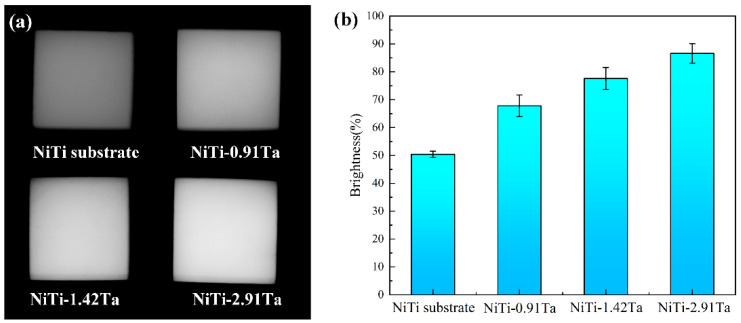
(**a**) X-ray images and (**b**) brightness of the NiTi substrate and NiTiTa coatings with different Ta contents.

Figure 12a presents the X-ray images of NiTiTa coatings and the NiTi substrate. It can be seen that the NiTi substrate has lower clarity, while the NiTiTa coatings are clearly visible, and the brightness increases with the increase in Ta content, which means that the shape and internal features of implants covered with the NiTiTa coating can be more easily detected. In addition, the X-ray images of NiTiTa coatings prove that the fabricated materials have no macroscopic defects, and Ta is uniformly distributed at the macroscopic scale.

In order to perform a quantitative analysis on the enhanced radiopacity of NiTiTa coatings with different Ta contents, the brightness was measured in HSB mode using the image processing software Photoshop CS6, 2021 and the results are shown in Figure 12b. The average brightness levels of the NiTi-0.91Ta, NiTi-1.42Ta, and NiTi-2.91Ta coatings are 67.8, 77.6, and 86.6%, respectively, while that of the NiTi substrate is only 50.4%. Since the X-rays used in this study generate images based on differences in attenuation after passing through materials, greater signal attenuation results in brighter images. Ta possesses a higher atomic number compared to Ni and Ti, which provides more extra-nuclear electrons to attenuate the penetration of X-rays [37]. The higher X-ray visibility makes the implantable devices coated with NiTiTa alloys easier to detect and track, showing better application prospects than NiTi alloys in biomedical applications.

## 4. Conclusions and Outlook

In this study, NiTiTa coatings with different Ta contents were successfully in situ-synthesized on a NiTi substrate through GTAW technology using commercially pure Ta foils as the feedstock material. NiTiTa coatings demonstrate promising prospects for medical applications and warrant further investigation in the medical field. The main conclusions are as follows:

(1) GTAW technology produced dense NiTiTa coatings with no observed pores, inclusions, or cracks. The roughness of the NiTiTa coatings was lower than that of the NiTi substrate, delivering good surface quality. An arc-shaped chimeric structure and a transitional interface with a thickness of 40 μm between the coating and the substrate were observed. The NiTiTa coatings were mainly composed of a large number of columnar grains, which grew along the heat dissipation direction.
(2) The NiTiTa coatings were mainly composed of B2 austenite and B19’ martensite, with (Ti, Ta)2Ni and β-Ta precipitating at the grain boundaries. The proportion of martensite, as well as the amount of β-Ta precipitation and the Ta content dissolved in the matrix, increased with the increasing Ta addition.
(3) The addition of Ta increased the corrosion potential and reduced the corrosion current density. However, excessive Ta led to the formation of more β-Ta phase, thereby diminishing the outstanding corrosion resistance.

Although this paper reports on the microstructure, hardness, corrosion resistance, and X-ray visibility of NiTiTa alloys, current research still has considerable room for improvement due to various constraints. For instance, in electrochemical corrosion processes, integrating electrochemical impedance spectroscopy can enhance the reliability of analytical results. Additionally, the absence of fatigue testing and in vitro biocompatibility assessments represents a significant shortcoming. Therefore, subsequent research should prioritize addressing these areas.

## Figures and Tables

**Table 1:** Chemical composition of raw material.

Materials	Composition, wt.%									
	Ni	C	O	N	H	Co	Cr	Cu	Ti	Ta
NiTi	55.58	-	0.032	0.004	0.00028	0.010	<0.010	<0.001	balanced	-
Ta foil	-	<0.001	0.005	0.002	0.0006	-	-	-	-	≥99.95

**Table 2:** Process parameters used for GTAW deposition.

Process Parameters	
Deposition current (A)	110
Travel speed (mm/min)	150
Distance from electrode to substrate (mm)	4
Pre-heating temperature (°C)	300
Trailing shield gas flow (L/min)	20
Torch nozzle gas flow (L/min)	15

**Table 3:** Sizes of Ta foils and corresponding Ta content in NiTiTa coatings.

Sample	Sizes of Ta Foils (Thickness × Width, mm)	Ta Content (at. %)
NiTi-0.91Ta	0.1 × 1.0	0.91
NiTi-1.42Ta	0.1 × 1.5	1.42
NiTi-2.91Ta	0.2 × 1.5	2.91

**Table 4:** Chemical compositions of the marked points of NiTiTa coatings with different Ta contents in Figure 6.

Points				Compositions (at.%)	
	Ti	Ni	Ta	(Ti + Ta)/Ni Ratio	Potential Phase
A	48.88	50.25	0.87	0.99	NiTi matrix
B	62.08	36.69	1.23	1.73	(Ti, Ta)_2_Ni
C	48.81	49.84	1.34	1.01	NiTi matrix
D	16.01	18.43	65.56	/	β-Ta
E	62.55	35.72	1.73	1.80	(Ti, Ta)_2_Ni
F	47.36	49.76	2.88	1.01	NiTi matrix
G	10.92	12.66	76.42	/	β-Ta
H	57.58	38.69	3.73	1.58	(Ti, Ta)_2_Ni

**Table 5:** The main electrochemical parameters.

	NiTi-0.91Ta	NiTi-1.42Ta	NiTi-2.91Ta	NiTi Substrate
***E_corr_*/mV (vs. SCE)**	−336.1 ± 14.2	−312.6 ± 13.6	−306.4 ± 15.9	−368.9 ± 16.3
***i_corr_***/10^−10^ **A·cm^−2^**	26.4 ± 6.2	36.6 ± 7.4	63.8 ± 12.1	65.9 ± 11.3

## Data Availability

The original contributions presented in this study are included in the article. Further inquiries can be directed to the corresponding author.
